# A Cardiovascular Risk Score for Use in Occupational Medicine

**DOI:** 10.3390/jcm10132789

**Published:** 2021-06-24

**Authors:** Giuseppina Affinito, Pasquale Arpaia, Francesco Barone-Adesi, Luca Fontana, Raffaele Palladino, Maria Triassi

**Affiliations:** 1 Department of Electrical Engineering and Information Technology, Federico II University of Naples, 80131 Naples, Italy; pasquale.arpaia@unina.it; 2 Department of Public Health, Federico II University of Naples, 80131 Naples, Italy; raffaele.palladino@unina.it (R.P.); triassi@unina.it (M.T.); 3 Interdepartmental Research Center in Healthcare Management and Innovation in Healthcare (CIRMIS), 80131 Naples, Italy; 4 Augmented Reality for Health Monitoring Laboratory (ARHeMLab), 80131 Naples, Italy; 5 Department of Translational Medicine, Università del Piemonte Orientale, 28100 Novara, Italy; francesco.baroneadesi@uniupo.it; 6 Research Center in Emergency and Disaster Medicine, Università del Piemonte Orientale (CRIMEDIM), 28100 Novara, Italy; 7 Department of Public Health, Section of Occupational Medicine, University of Naples Federico II, 80131 Naples, Italy; luca.fontana@unina.it; 8 Department of Primary Care and Public Health, Imperial College of London, London W6 8RP, UK

**Keywords:** cardiovascular risk, public health, occupational medicine, work

## Abstract

Cardiovascular disease is one of the most frequent causes of long-term sickness absence from work. The study aims to develop and validate a score to assess the 10-year risk of unsuitability for work accounting for the cardiovascular risk. The score can be considered as a prevention tool that would improve the cardiovascular risk assessment during health surveillance visits under the assumption that a high cardiovascular risk might also translate into high risk of unsuitability for work. A total of 11,079 Italian workers were examined, as part of their scheduled occupational health surveillance. Cox proportional hazards regression models were employed to derive risk equations for assessing the 10-year risk of a diagnosis of unsuitability for work. Two scores were developed: the $CROM_{A}$ score (Cardiovascular Risk in Occupational Medicine) included age, sex, smoking status, blood pressure (systolic and diastolic), body mass index, height, diagnosis of hypertension, diabetes, ischemic heart disease, mental disorders and prescription of antidiabetic and antihypertensive medications. The $CROM_{B}$ score was the same as $CROM_{A}$ score except for the inclusion of only variables statistically significant at the 0.05 level. For both scores, the expected risk of unsuitability for work was higher for workers in the highest risk class, as compared with the lowest. Moreover results showed a positive association between most of cardiovascular risk factors and the risk of unsuitability for work. The $CROM_{A}$ score demonstrated better calibration than the $CROM_{B}$ score (11.624 (*p*-value: 0.235)). Moreover, the $CROM_{A}$ score, in comparison with existing CVD risk scores, showed the best goodness of fit and discrimination.

## 1. Introduction

Cardiovascular disease (CVD) is the most common cause of death globally, with 17.9 million deaths estimated each year. Of these deaths, 85% are due to cerebrovascular diseases, and one third of these deaths occur in people under 70 years of age [1,2]. CVD is a major cause of disability for all age-groups worldwide, including working-age individuals [3]. Moreover, several occupational risk factors including work-related stress, long working hours or manual handling of heavy loads are associated with increased risk of CVD [4,5]. The hypothesized mechanism between long working hours and cardiovascular health is related to reduced time available for other activities besides work. Employees working long hours spend more time at the workplace and thus they might be increasingly exposed to psycho–social, chemical and/or physical occupational risk factors which could adversely impact the cardiovascular system [6] or, on the other hand, workers engaged in performing sedentary jobs (i.e., office work) have reduced physical activity and increased CVD risk [7]. Obviously, these associations might be stronger for specific job categories, which involve exposure to occupational risk factors whose damaging action can mainly affect the cardiovascular system but, to certain extent, they apply for all types of jobs [4,5]. Furthermore, CVD may negatively impact productivity and quality of work [8]. CVD is one of the most frequent causes of long-term sickness absence from work. Additionally, the illness consequences may cause a radical change of work activity [9], which prompts the question of whether a thorough cardiovascular risk assessment and appropriate preventive strategies are appropriately integrated as part of the occupational health surveillance. The majority of existing cardiovascular risk scores in the literature have been adopted in medical settings different from occupational medicine [10,11,12,13,14,15,16]. These scores have been created to evaluate the risk of diagnosis cardiovascular diseases or risk of mortality due to CVD either in a general population or in a certain occupational cohort (i.e., male industrial workers or government officials) [7,17,18]. Interestingly, previous research recalibrated the Framingham risk score to evaluated the risk of unsuitability for work in a cohort of Italian workers without, however, obtaining clinically meaningful results [3]. Therefore, in this context, this study aims is to derive, calibrate, and validate a new score to predict the risk of unsuitability for work, accounting for the cardiovascular risk factors. Specifically, the study aims to provide a prevention tool to improve the cardiovascular risk assessment during health surveillance visits under the assumption that a high cardiovascular risk might also translate into a high risk of unsuitability for work. The score, named Cardiovascular Risk in Occupational Medicine (CROM), was also compared with CVD risk scores already present in the literature.

## 2. Materials and Methods

### 2.1. Study Design and Study Population

Th study is a retrospective cohort study using data from a cohort of 11,079 workers. Workers were employed by the Naples municipalities and were examined as part of their scheduled occupational health surveillance, at the occupational health outpatient clinic of the Department of Public Health of the University “Federico II” of Naples between January 2006 and December 2016. All clinical assessments were part of clinical practice in a university setting and data were fully anonymized. All subjects signed the general informed consent form, authorizing the use of observational clinical data for research purposes.

### 2.2. Study Variables

Data collection was performed during a medical examination for scheduled occupational health surveillance. According to the Italian occupational medicine legislation and above all considering the different levels and types of occupational and/or job strain, workers were classified into four groups, which translated into a different periodicity of their scheduled occupational health surveillance visit (every one/two/three/five years) [19]. A detailed list of each job included in this analysis can be found in the Appendix A. At the end of each visit for health surveillance, according to Italian occupational medicine guidelines, a fitness for work judgment was issued by the occupational medicine physician and later confirmed by a senior occupational medicine consultant. The fitness for work judgment has three possible outcomes: (i) suitability; (ii) suitability with limitations or prescriptions (with a consequent reduction or remodeling of the job strain for the worker); (iii) total unsuitability, with a radical change of activities within the job. Incident diagnosis of unsuitability for work (both partial with limitations or prescriptions and total) over a 10-year follow-up period was considered as study outcome. As part of the medical examination, socio-demographic and clinical history data, as well as laboratory and instrumental data, were collected. Study covariates included age, sex, smoking status, blood pressure (systolic and diastolic), body mass index (BMI), height, diagnosis of hypertension, diagnosis of diabetes, Ischemic heart disease, mental disorders (bipolar disorder and moderate/severe depression) and prescription of drugs, especially anti-diabetic medications (biguanides, sulfonylureas, insulin, others), anti-hypertensive (ACE inhibitor (ACEi) or angiotensin receptor blocker (ARB); others). Additionally, taking into account the considerable heterogeneity of the work carried out by the study participants, in order to consider the different exposures (and the relative extent) to occupational risk factors and job strain impacting on the cardiovascular system, we have introduced a new variable “job risk class” (highest risk class, medium-high risk class, medium-low risk class and lowest risk class) based on the health surveillance protocol adopted which, in turn, is established on the basis of the type and level of exposure to occupational risk factors [3]. On the contrary, a categorical variable describing each job was not included due to sample size constraints. Analysis of variables was conducted at baseline, considering only the first visit for each individual. For each covariate in the study, baseline values were considered. To reduce the amount of missing data at baseline for study covariates, we used the first available clinical recording within five years for each individual for individuals [20]. Cigarette smoking status was ascertained by self-reporting. The individuals with missing data on smoking were classified as non-smokers. Similarly, the absence of recording for CVD was considered as the absence of the condition [3,20,21].

### 2.3. Statistical Analysis

Missing data were present for the following variables: body mass index (3.25% missing), systolic blood pressure (2.67% missing), diastolic blood pressure (2.66% missing), height (3.25% missing). We used multiple imputations by chained equations to replace missing values for body mass index, systolic blood pressure, diastolic blood pressure and height [22,23]. Predictor variables to include in the imputation models were identified employing a missing-pattern analysis that used a multivariable logistic regression model to assess factors associated with missing data. We included sex and diagnosis of hypertension variables in the imputation model as likely to be associated with the recording of risk factor. We carried out five imputations, as this has relatively high efficiency and was a pragmatic approach accounting for the size of the data set and capacity of the available servers and software [13]. We used the Rubin rule to combine the results across the imputed data set. Dir references in covariate distribution between occupational risk groups were explored employing ANOVA and χ-squared, as appropriate. Variables such as sex and age were considered, but they were not statistically significant [10]. We implemented Cox proportional hazards regressions to predict the risk of a diagnosis of unsuitability over a 10-year follow-up period (Appendix C). To build the multivariate Cox proportional hazard regression model, for the model derivation a model-building strategy was employed. Similarly to previous research [20], the approach was based on evaluating both the statistical significance and clinical relevance of the variables to ensure those candidate variables were likely to be clinically important and to reduce the over-fitting and optimism of the model. First, a multivariate Cox proportional hazard regression model including all candidate independent variables was run. In line with previous research [3,19,23], variables were included in the multivariate model if they had a hazard ratio of less than 0.90 or more than 1.10 (for binary variables) and were statistically significant at the 0.01 level in the univariate analyses. Although not significant, age and sex were considered because they were likely to be associated with the outcome [10]. Interactions between predictor variables and age at baseline were also examined and then significant interactions were included in the final models. All the continuous variables were naturally logarithmically transformed to improve discrimination and calibration of the models and to minimize the influence of extreme observations [10]. In addition, a multivariate Cox proportional hazard regression model using data where cases with missing data are excluded was run (Appendix B). Model discrimination was evaluated through an interval validation employing a k Fold Cross-Validation procedure and the C-statistic’s measure [24]. Harrell’s C statistic is a goodness of fit measure for models which produce risk scores and values approaching 1 indicate a good model.

The calibration of the validated risk score, a measure of agreement between observed and predicted events, was evaluated using the Gronnesby and Borgan Test based on martingale residuals [25]. The output returns a chi-square value (chi-squared) and a *p*-value (e.g., Pr > ChiSq). Small p-values mean that the model is a poor fit.

Using the person’s absolute risk [26], empirical research on the risk threshold was undertaken, as previously suggested [27]. Finally, the validated score was compared with the widely used CVD risk scores. Three CVD risk scores already present in the literature (the Framingham score, European SCORE and Q-risk score) were recalibrated employing Cox proportional hazard regression models. Specifically, one model included risk factors present in the Framingham score (age, sex, smoking status, systolic blood pressure and antihypertensive), a second model included the risk factors present in the European SCORE (age, sex, smoking status, systolic blood pressure, body mass index (BMI)) and a third model was developed using risk factors present in the Q-risk score (age, sex, smoking status, systolic blood pressure, body mass index (BMI), diagnosis of diabetes, diagnosis of Ischemic heart disease, anti-hypertensive, diagnosis of mental disorders) [12,28].

Since the cohort used in the study is a healthy working age cohort, the coefficients of unavailable covariates were kept constant and equal to 1 under the assumption that no recording meant absence of the condition.

## 3. Results

Between January 2006 and December 2016, 11,079 workers were examined for health surveillance by trained physicians at the Occupational Medicine Outpatient Clinic of “Federico II” University Hospital. A total of 57.95% were men, the mean (SE) age was 52.35 (8.46), 6.31% were in the highest risk class, 53.06% were in the medium–high risk Class, 32.85% were in the medium–low risk Class and 7.79% were in the lowest risk class. The statistical description of the variables after multiple imputations is shown in Table 1.

Table 2 and Table 3 show the adjusted hazard ratios for the $CROM_{A}$ score and the $CROM_{B}$ score. The $CROM_{A}$ score included all the variables above; the $CROM_{B}$ score was the same as the $CROM_{A}$ score except that it included only variables that were statistically significant at the 0.05 level in the Multivariable Cox Model.

The number of events of unsuitability for work was sufficiently high to allow the risk score derivation. In fact, 852 events (i.e., 852 diagnoses of unsuitability for work) were recorded.

The results show a positive association between most of the cardiovascular risk factors and the risk of unsuitability for work. Moreover, the tables (Table 2 and Table 3) show a positive proportional link between the worker risk classes and the risk of unsuitability for work. Moreover, the estimated regression coefficient increases with the level of exposure to occupational risk factors and so the risk of unsuitability for work gradually decreased with decreasing level of exposure to occupational risk factors.

**Figure 1:**
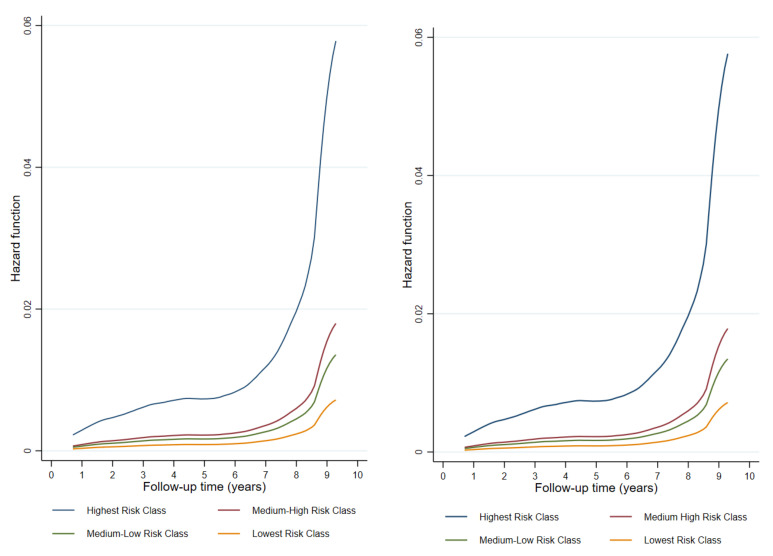
Hazard Function: Trend of the hazard function as a function of analysis time (10 years) depending on occupational risk classes. On the right, the performance of hazard function using the $CROM_{A}$ score, on the left, the performance of hazard function using the $CROM_{B}$ score.

It possible to observe this result also in Figure 1, where the hazard function value increases with both follow-up time and with the level of exposure to occupational risk factors (highest risk class, medium–high risk class, medium–low risk class and lowest risk class).

In addition to measures of discrimination of the model, for both scores $CROM_{A}$ and $CROM_{B}$, we calculated Harrell’s C statistics. The result for the $CROM_{A}$ score was 0.700 (95% CI: 0.698–0.702), and was 0.699 (95% CI: 0.696, 0.702) for the $CROM_{B}$ score.

**Figure 2:**
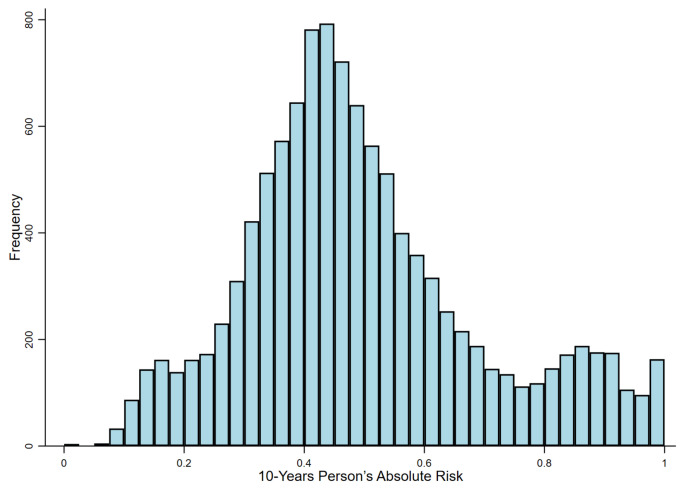
Absolute risk: The histogram shows the person’s absolute risk calculated for the study cohort using the $CROM_{A}$ score. About 67% of workers present a cumulative risk larger than or equal to 0.4; therefore, the risk of 40% appears to be a good threshold for the discrimination of high-risk workers.

For the calibration assessment, the $CROM_{A}$ score chi-square was 11.624 (*p*-value: 0.235) whilst was the chi-square for the $CROM_{B}$ score was 11.000 (*p*-value: 0.275); therefore, the $CROM_{A}$ score fitted better than the $CROM_{B}$ score to the set of observations. Comparing the two scores developed in the study, the $CROM_{A}$ score had better calibration and discrimination. Figure 2 shows the person’s absolute risk of unsuitability for work [10,26] for the study cohort using the $CROM_{A}$ score. For over 60% of cases, the status of unsuitability for work occurred for a risk greater than or equal to 46.2%; therefore, the risk of 40% appears to be a good threshold for discrimination of high-risk workers.

We calculated Harrell’s C statistics for widely used CVD risk scores. The result for the Framingham score was 0.581 (95% CI: 0.577, 0.585), for the European score it was 0.590 (95% CI: 0.588, 0.593) and for the Q-risk score it was 0.608 (95%CI: 0.609, 0.612). For the calibration assessment, the Framingham score’s chi-square was 13.512 (*p*-value: 0.140), the European score’s chi-square was 14.126 (*p*-value: 0.117) and the Q-risk score’s chi-square was 18.592 (*p*-value: 0.028). The $CROM_{A}$ score, in comparison with the used CVD risk scores above, had the best goodness of fit and discrimination.

## 4. Discussion

Using data from a cohort of 11,079 workers with different jobs, this study derived and validated a new risk score to evaluate the 10-year risk of a diagnosis of unsuitability for work due to cardiovascular diseases. The equation incorporates canonical cardiovascular risk factors as well as new risk factors associated with the workplace. Our findings confirm the linkage between the CVD risk factor and the workplace [2,7,17]. The proportion of individuals with a diagnosis of unsuitability for work grows progressively from the lowest to the highest work risk classes. Many CVD risk scores have been derived and are currently used in clinical practice. However, those scores have been designed to be used on the general population and might show little accuracy when applied to specific populations like the working-age population.

In our opinion, the use of CROM score could have significant practical implications in the field of occupational medicine, especially in implementing the preventive potential of health surveillance. Indeed, occupational medicine is basically a preventive specialty and occupational physicians have a fundamental role in protecting and improving the health of employees in relation to their work and to ensure a continual improvement of working environment and preventive and/or protective measures [29,30]. Therefore, in this context, a high CROM score (predictive of a likely unsuitability of the worker in the medium–long term) should not be used to possibly anticipate such unsuitability (this would not be correct from an occupational medicine point of view and not even acceptable from an ethical perspective as, when the score is calculated, it is likely that the worker is completely fit to perform his work), but rather it should represent an alarm bell for the occupational physician but, more generally, the entire occupational safety and health management system should therefore undertake to apply specific and appropriate additional preventive measures to protect the worker with a high CROM score’s health. Obviously, the preventive strategy that is decided upon can be put into practice by exploiting a wide range of tools whose use should be integrated and in any case designated on the basis of occupational risk factors (which in turn depend on the type of work performed) to which the worker is exposed to. In other words, a high CROM value, let’s say for example, to a worker who carries out the activity of warehouseman and who, in order to carry out his job tasks, is exposed to manual handling of heavy loads, night work and working activities that involve energy cost superior to six metabolic equivalents of the task, could be suggested the opportunity to reshape workloads or implement the health surveillance protocol (carrying out additional blood tests). However, on the other hand, the same CROM value, determined in a completely different type of worker such as an office worker, exposed only to a video display unit, would have quite different practical implications. Indeed, in this case, considering the low impact of exposure to occupational risk factors on the cardiovascular system, the main task of the occupational physician should be to carry out an adequate counseling action (i.e., health promotion) to reduce overall CVD risk factors (eventually including work-related ones such as psychosocial factors). However, differences in accuracy might also be explained by the fact that other scores were derived using not only different populations but also considering different outcomes. In addition, the score showed good discrimination power (0.700176 95% CI:0.700, 0.780), considering that similar levels from different scores have already been accepted when implementing prevention tools in a similar setting [7,31]. The majority of cardiovascular risk scores were developed to assess the CVD risk using the whole population and including accepted cardiovascular risk factors associated with [7,10,12,27]. Previous research has only occupational stress or job strain with which to assess the link between CVD risk factors and workplace [7,32]. Therefore, using only psychological job demand and occupational stressors, the job conditions were not examined in their entirety. To our knowledge, this is the first study that aimed to assess the risk of unsuitability for work adjusted for cardiovascular risk factors using a heterogeneous cohort of workers and a long follow-up time. In addition, this is also the first time that the job conditions were weighed by assessing both occupational risks (chemical, physical and biological risks) and job strain. While most studies consider mental health to be the leading cause of unsuitability for work, we included several mental disorders in a single covariate, minimizing its weight [33,34,35]. Several caveats merit discussion. Based on data availability, it was not possible to classify diagnoses of unsuitability for work according to whether it was CVD-related. Whilst this might be considered as a main limitation, many studies have shown that diagnosis of unsuitability for work is often multifactorial and involves many risk factors, including CVD risk factors which are likely to have contributed to it [36]. However, future studies should be conducted to externally validate the CROM score using datasets including information on reason for diagnosis of unsuitability for work.

Additional study limitations include the presence of missing data for clinical variables such as body mass index, systolic blood pressure, diastolic blood pressure, and height. However, we overcame the latter issue by using multiple imputations by chained equations.

A further important limitation of the study is the absence in the literature of a score that can be comparable to the CROM score due to different outcomes considered in each score and the model derivation carried out using different populations and settings. However, given that the CROM score should not be used to possibly anticipate unsuitability for work but as support to apply specific preventive measures, we overcame this issue by recalibrating three CVD risk scores already present in the literature employing Cox proportional hazard regression models to make them comparable with the CROM score (the Framingham score, European SCORE and Q-risk score).

Another limitation of the study includes the use of a limited kind of job activity; in fact, only those available to us have been used and so are present in the dataset. The existing literature supports the ineffectiveness of models derived and validated on a specific ethnic group when translated to a different one [16]. Therefore, external validation should be conducted to estimate the model predictiveness and validity on a more heterogeneous cohort including other ethnic groups as well and a broader range of jobs.

### Public Health and Occupational Medicine Implications

CVD is one of the most prevalent causes of long sickness absence from work and may involve the radical change of individual work activities. Therefore, the score developed in this work could be used at each visit of health surveillance as a clinical tool to predict the 10-year risk of unsuitability for work using the personal information and medical history of the worker. Considering that high cardiovascular risk is also associated with a higher risk of unsuitability for work and that an occupational medicine doctor might have limited knowledge of the worker’s medical history, it might be useful to have a tool with which to conduct an accurate assessment of the risk of unsuitability for work due to cardiovascular factors and to adopt non-pharmaceutical and pharmaceutical interventions to reduce it [37].

A modern occupational physician is a leading expert on mitigating the impact of health conditions on workers’ professional activity. Therefore, in order to achieve this important goal, occupational physicians should not only consider possible workplace-related threats to workers’ health but they should also take into account any diseases, health issues, disabilities or risk factors that might be an obstacle to the adequate and safe performance of job activities [38]. In this regard, the management of workers with high CVD risk scores is a rather challenging issue which necessarily requires evaluating the complex interplay between CVD risk factors and exposure to occupational risk factors impacting the cardiovascular system. In the health surveillance context, the use of the CROM score could be useful, since it is an integrated and comprehensive tool. This would provide occupational physician with interesting data to ensure adequate safe and healthy working conditions.

## 5. Conclusions

We derived and validated an accurate score to predict the 10-year risk of unsuitability for work in occupational medicine. This score could be used as a preventive strategy clinical tool for cardiovascular risk assessment during the scheduled medical examination for health surveillance to reduce cardiovascular risk and so reduce the probability of unsuitability for work associated with cardiovascular risk factors.

## Figures and Tables

**Table 1:** Baseline characteristics of workers. Notes: IHD indicates Ischemic heart disease.

Continuous Covariates	Mean	SD
Age	52.35	8.46
Height	1.66	0.17
Systolic Blood Pressure	126.78	16.02
Diastolic Blood Pressure	80.30	9.16
**Discrete Covariates**	**Freq (N = 11,079)**	**Perc**
Female	4659	42.05%
Male	6420	57.60%
Smoker	4609	41.60%
Diabetes	474	4.28%
IHD	350	3.167%
Cerebral Ischemia	10	0.09%
Stroke	30	0.27%
Valvulopathy	113	1.02%
Bipolar Disorder	3	0.03%
Hypertension	2128	19.21%
Anxiety–Depressive Disorder	199	1.80%
Anti-hypertensive	1708	15.42%
Oral Blood Glucose Lowering	330	2.98%
Highest Risk Class	699	6.31%
Medium–High Risk Class	5878	53.06%
Medium–Low Risk Class	3639	32.85%
Lowest Risk Class	863	7.79%

**Table 2:** Regression Coefficients and Hazard Ratios of the $CROM_{A}$ score. Notes: * Estimated Regression Coefficients.

Covariates	$b{\;}^{*}$	Hz. Ratio	*p*-Value	95% CI
Sex (Male)	−0.376	0.687	<0.001	(0.564; 0.836)
ln (Age${}^{2}$)	0.435	1.544	0.007	(1.125; 2.117)
ln (Height)	−2.394	0.091	0.008	(0.015; 0.541)
ln (Systolic Blood Pressure)	1.063	1.136	0.719	(0.568; 2.273)
ln (Diastolic Blood Pressure)	0.127	2.894	<0.001	(1.633; 5.127)
ln (Body Max Index)	0.564	1.758	0.006	(1.175; 2.628)
Smoker	0.159	1.173	0.021	(1.024; 1.342)
Diabetes	0.507	1.661	0.002	(1.201; 2.297)
Ischemic Heart Disease	0.924	2.520	<0.001	(1.983; 3.203 )
Ischemia	1.469	4.346	0.004	(1.613; 11.708)
Stroke	0.773	2.167	0.051	(0.997; 4.711)
Valvupathy	1.026	2.791	<0.001	(2.048; 3.804)
Bipolar Disorder	1.504	4.500	0.014	(1.349; 15.009)
Hypertension	−0.347	0.706	0.002	(0.567; 0.879)
Anxiety–Depressive Disorder	0.819	2.270	<0.001	(1.629; 3.162)
Antihypertensive	0.337	1.401	0.008	(1.093; 1.796)
Hypoglycaemic	−0.083	0.920	0.699	(0.603; 1.403)
Highest Risk Class	2.118	8.317	<0.001	(4.130; 15.613)
Medium–High Risk Class	0.924	2.521	0.003	(1.363; 4.661)
Medium–Low Risk Class	0.639	1.896	0.043	(1.020; 3.521)

**Table 3:** Regression Coefficients and Hazard Ratio of the $CROM_{B}$ score. Notes: * Estimated Regression Coefficients.

Covariates	$b{\;}^{*}$	Hz. Ratio	*p*-Value	95% CI
Sex (Male)	−0.372	0.689	<0.001	(0.566; 0.838)
ln (Age)	0.875	2.400	0.007	(1.277; 4.510)
ln (Height)	−2.396	0.091	0.008	(0.015; 0.539)
ln (Diastolic Blood Pressure)	0.121	2.892	<0.001	(1.633; 5.121)
ln (Body Max Index)	0.545	1.726	0.008	(1.154; 2.583)
Smoker	0.158	1.172	0.021	(1.024; 1.341)
Diabetes	0.474	1.607	<0.001	(1.278; 2.019)
Ichemic Heart Disease	0.935	2.547	<0.001	(2.007; 3.230)
Ischemia	1.464	4.325	0.004	(1.606; 11.649)
Valvulopathy	1.015	2.761	<0.001	(2.026; 3.762)
Bipolar Disorder	1.513	4.540	0.014	(1.361; 15.134)
Hypertension	−0.324	0.723	0.003	(0.582; 0.897)
Anxiety–Depressive Disorder	0.817	2.263	<0.001	(1.626; 3.148)
Antihypertensive	0.313	1.368	0.012	(1.070; 1.747)
Highest Risk Class	2.118	8.322	<0.001	(4.432; 15.623)
Medium–High Risk Class	0.922	2.515	0.003	(1.359; 4.650)
Medium–Low Risk Class	0.637	1.891	0.044	(1.018; 3.512)

**Figure A1:**
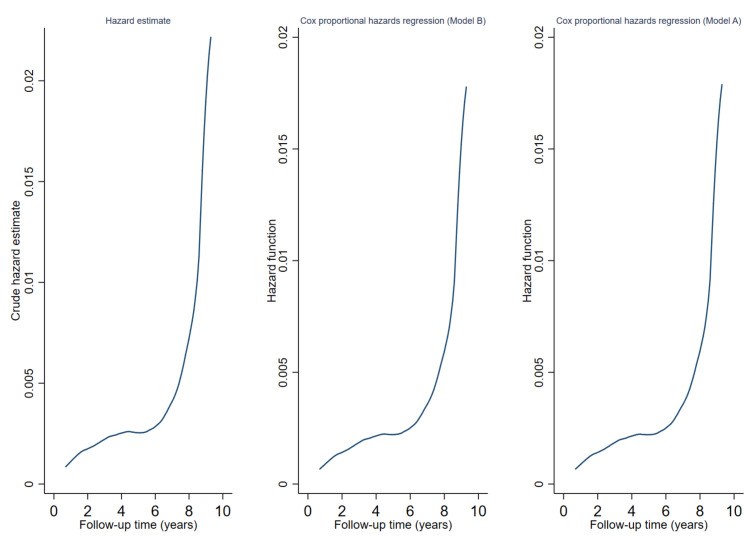
Calibration: Trend of hazard functions of both the CROM${}_{A}$ and CROM${}_{B}$ models after Cox regression versus the crude hazard estimate. The trends are similar, so there is a good fit of estimated data to observed data.

## Data Availability

The data presented in this study are available on request from the corresponding author. The data are not publicly available.

## Appendix A

Job risk factors, according to Italian legislation on occupational medicine, are classified into four groups. The groups reflect the workers’ risks (physical, chemical and biological risks) levels and they translate into a different periodicity of the scheduled visit to monitor health at work (every one/two/three/five years). Table A1 shows a detailed list of each job included in this analysis where the occupational risk group is composed of jobs with a similar surveillance protocol and therefore a similar health surveillance visit scheduling.

**Table A1:** List of jobs and their classification into four occupational risk groups (I as the highest, IV as the lowest).

Occupational Risk Group	Description of the Job	Periodicity of Heath Surveillance Visit
1	CLEANER	every year
1	DRIVER	every year
1	SEWER WORKER	every year
1	CEMETERY WORKER	every year
1	ELECTRICIAN	every year
1	MECHANIC	every year
1	COOK	every year
1	LAUNDRY SERVICE	every year
2	COORDINATOR CLEANING SERVICE	every two years
2	STAFF COORDINATOR	every two years
2	SEWER WORKERS COORDINATOR	every two years
2	WATCHMAN—DAILY HOURS	every two years
2	GARDNER	every two years
2	TRAFFIC OFFICER	every two years
2	OFFICE WORKER–PHOTOCOPIER	every two years
2	TEACHER—NURSERY SCHOOL	every two years
3	SOCIAL WORKER	every three years
3	SCHOOL JANITOR	every three years
3	MUSEUM CUSTODIAN—DAILY HOURS	every three years
3	MUSEUM GUIDE	every three years
3	OFFICE WORKER	every three years
3	TEACHER	every three years
4	VISUAL DISPLAY UNIT OPERATOR	every five years

## Appendix B

In addition to analysing that described above, a multivariate Cox proportional hazard regression model using data where cases with missing data are excluded was run. The results of the two analyses turned out to be comparable (Table A2 and Table A3).

**Table A2:** Regression Coefficients and Hazard Ratios of the $CROM_{A}$ score using complete-case data for body mass index, systolic blood pressurte, diastolic blood pressure and height. Notes: * Estimated Regression Coefficients.

Covariates	$b{\;}^{*}$	Hz. Ratio	*p*-Value	95% CI
Sex (Male)	−0.376	0.687	<0.001	(0.564; 0.836)
ln (Age${}^{2}$)	0.435	1.544	0.007	(1.125; 2.117)
ln (Height)	−2.394	0.091	0.008	(0.015; 0.541)
ln (Systolic Blood Pressure)	1.063	1.136	0.719	(0.568; 2.273)
ln (Diastolic Blood Pressure)	0.127	2.894	<0.001	(1.633; 5.127)
ln (Body Mass Index)	0.564	1.758	0.006	(1.175; 2.628)
Smoker	0.159	1.173	0.021	(1.024; 1.342)
Diabetes	0.507	1.661	0.002	(1.201; 2.297)
Ischemic Heart Disease	0.924	2.520	<0.001	(1.983; 3.203 )
Ischemia	1.469	4.346	0.004	(1.613; 11.708)
Stroke	0.773	2.167	0.051	(0.997; 4.711)
Valvupathy	1.026	2.791	<0.001	(2.048; 3.804)
Bipolar Disorder	1.504	4.500	0.014	(1.349; 15.009)
Hypertension	−0.347	0.706	0.002	(0.567; 0.879)
Anxiety–Depressive Disorder	0.819	2.270	<0.001	(1.629; 3.162)
Antihypertensive	0.337	1.401	0.008	(1.093; 1.796)
Hypoglycaemic	−0.083	0.920	0.699	(0.603; 1.403)
Highest Risk Class	2.118	8.317	<0.001	(4.130; 15.613)
Medium–High Risk Class	0.924	2.521	0.003	(1.363; 4.661)
Medium–Low Risk Class	0.639	1.896	0.043	(1.020; 3.521)

**Table A3:** Regression Coefficients and Hazard Ratios of the $CROM_{B}$ score using complete-case data for body mass index, systolic blood pressurte, diastolic blood pressure and height. Notes: * Estimated Regression Coefficients.

Covariates	$b{\;}^{*}$	Hz. Ratio	*p*-Value	95% CI
Sex (Male)	−0.372	0.689	<0.001	(0.566; 0.838)
ln (Age)	0.875	2.400	0.007	(1.277; 4.510)
ln (Height)	−2.396	0.091	0.008	(0.015; 0.539)
ln (Diastolic Blood Pressure)	0.121	2.892	<0.001	(1.633; 5.121)
ln (Body Max Index)	0.545	1.726	0.008	(1.154; 2.583)
Smoker	0158	1.172	0.021	(1.024; 1.341)
Diabetes	0.474	1.607	<0.001	(1.278; 2.019)
Ichemic Heart Disease	0.935	2.547	<0.001	(2.007; 3.230)
Ischemia	1.464	4.325	0.004	(1.606; 11.649)
Valvulopathy	1.015	2.761	<0.001	(2.026; 3.762)
Bipolar Disorder	1.513	4.540	0.014	(1.361; 15.134)
Hypertension	−0.324	0.723	0.003	(0.582; 0.897)
Anxiety–Depressive Disorder	0.817	2.263	<0.001	(1.626; 3.148)
Antihypertensive	0.313	1.368	0.012	(1.070; 1.747)
Highest Risk Class	2.118	8.322	<0.001	(4.432; 15.623)
Medium–High Risk Class	0.922	2.515	0.003	(1.359; 4.650)
Medium–Low Risk Class	0.637	1.891	0.044	(1.018; 3.512)

Calibration assessment of the CROM score was performed using the Gronnesby and Borgan Test based on martingale residuals. The CROM${}_{A}$ and CROM${}_{B}$ models present a good calibration, which was confirmed also by Figure A1, showing a good fit to the crude hazard estimate for both models.

## Appendix C

### Appendix Materials and Methods

Cox proportional hazards regression to predict the risk of a diagnosis of unsuitability for work during a follow-up period of 10 years was implemented:

$$h\left(t\right)=h_{o}\left(t\right)e^{\left(b_{0}x_{0}+b_{1}x_{1}+b_{i}x_{i}+\ldots +b_{N}x_{N}\right)}$$

where h(t) is the hazard function and it is defined as the instantaneous risk that a worker is evaluated as unsuitability for work, within a very narrow time frame. $h_{0}\left(t\right)$ is the baseline or underlying hazard function and corresponds to the probability of unsuitability for work when all the explanatory variables are zero.

In order to choose covariates to include in Cox’s multi-variables regression model, for each risk factor, the Hazard Ratio of Cox’s uni-variable model was evaluated.

$$HR=\frac{h(t)}{h_{0}\left(t\right)}=e^{\left(b_{i}x_{i}\right)}$$

Variables that had a hazard ratio of less than 0.90 or more than 1.10 (for binary variables) and were statistically significant at the 0.01 level were included in the model.

An assessment test for multicollinearity in both CROM models was run. Moreover, we tested for interactions between each variable and age. However, these interactions were not included in the final model because they were not significant.

## References

[1] Cardiovascular Diseases. https://www.who.int/health-topics/cardiovascular-diseases/#tab=tab_1.

[2] Cardiovascular Diseases (CVDs). https://www.who.int/news-room/fact-sheets/detail/cardiovascular-diseases-(cvds).

[3] Palladino R., Caporale O., Nardone A., Fiorentino D., Torre I., Triassi M. (2016). Use of Framingham Risk Score as a Clinical Tool for the Assessment of Fitness for Work: Results From a Cohort Study. J. Occup. Environ. Med..

[4] Kivimäki M., Kawachi I. (2015). Work Stress as a Risk Factor for Cardiovascular Disease. Curr. Cardiol. Rep..

[5] Torquati L., Mielke G.I., Brown W.J., Kolbe-Alexander T. (2018). Shift work and the risk of cardiovascular disease. A systematic review and meta-analysis including dose—Response relationship. Scand. J. Work. Environ. Health.

[6] Virtanen M., Kivimäki M. (2018). Long Working Hours and Risk of Cardiovascular Disease. Curr. Cardiol. Rep..

[7] Veronesi G., Borchini R., Landsbergis P., Iacoviello L., Gianfagna F., Tayoun P., Grassi G., Cesana G., Ferrario M.M., Ferrario M.M. (2018). Cardiovascular disease prevention at the workplace: Assessing the prognostic value of lifestyle risk factors and job-related conditions. Int. J. Public Health.

[8] Price A.E. (2004). Heart disease and work. Heart.

[9] Ármannsdóttir B., Mårdby A.C., Haukenes I., Hensing G. (2013). Cumulative incidence of sickness absence and disease burden among the newly sick-listed, a cross-sectional population-based study. BMC Public Health.

[10] D’agostino R.B., Vasan R.S., Pencina M.J., Wolf P.A., Cobain M., Massaro J.M., Kannel W.B. (2008). General Cardiovascular Risk Profile for Use in Primary Care. Circulation.

[11] Stern M.P., Williams K., González-Villalpando C., Hunt K.J., Haffner S.M. (2004). Does the Metabolic Syndrome Improve Identification of Individuals at Risk of Type 2 Diabetes and/or Cardiovascular Disease?. Diabetes Care.

[12] Conroy R.M., Pyörälä K., Fitzgerald A.E., Sans S., Menotti A., De Backer G., De Bacquer D., Ducimetiere P., Jousilahti P., Keil U. (2003). Estimation of ten-year risk of fatal cardiovascular disease in Europe: The SCORE project. Eur. Heart J..

[13] Hippisley-Cox J., Coupland C. (2017). Development and validation of QDiabetes-2018 risk prediction algorithm to estimate future risk of type 2 diabetes: Cohort study. BMJ.

[14] Basu S., Sussman J.B., Berkowitz S.A., Hayward R.A., Yudkin J.S. (2017). Development and validation of Risk Equations for Complications Of type 2 Diabetes (RECODe) using individual participant data from randomised trials. Lancet Diabetes Endocrinol..

[15] Basu S., Sussman J.B., Berkowitz S.A., Hayward R.A., Bertoni A.G., Correa A., Mwasongwe S., Yudkin J.S. (2018). Validation of Risk Equations for Complications of Type 2 Diabetes (RECODe) Using Individual Participant Data From Diverse Longitudinal Cohorts in the U.S. Diabetes Care.

[16] Gray L.J., Taub N.A., Khunti K., Gardiner E., Hiles S., Webb D.R., Srinivasan B.T., Davies M.J. (2010). The Leicester Risk Assessment score for detecting undiagnosed Type 2 diabetes and impaired glucose regulation for use in a multiethnic UK setting. Diabet. Med..

[17] Mirmohammadi S.J., Taheri M., Mehrparvar A.H., Heydari M., Kanafi A.S., Mostaghaci M. (2014). Occupational stress and cardiovascular risk factors in high-ranking government officials and office workers. Iran. Red Crescent Med. J..

[18] Harari G., Green M.S., Zelber-Sagi S. (2017). Estimation and development of 10- and 20-year cardiovascular mortality risk models in an industrial male workers database. Prev. Med..

[19] (2008). Italian Department of Welfare and Labour. d. lgs. 9 Aprile. http://www.cip.srl/documenti/Testo%20Unico%20Salute%20e%20Sicurezza%20sul%20lavoro%20-%20D.lgs.%2081-2008.pdf.

[20] Palladino R., Tabak A.G., Khunti K., Valabhji J., Majeed A., Millett C., Vamos E.P. (2020). Association between pre-diabetes and microvascular and macrovascular disease in newly diagnosed type 2 diabetes. BMJ Open Diabetes Res. Care.

[21] Chang K.C.M., Vamos E.P., Palladino R., Majeed A., Lee J.T., Millett C. (2019). Impact of the NHS Health Check on inequalities in cardiovascular disease risk: A difference-in-differences matching analysis. J. Epidemiol. Community Health.

[22] Steyerberg E.W., van Veen M. (2007). Imputation is beneficial for handling missing data in predictive models. J. Clin. Epidemiol..

[23] Moons K.G.M., Donders R.A.R.T., Stijnen T., Harrell F.E. (2006). Using the outcome for imputation of missing predictor values was preferred. J. Clin. Epidemiol..

[24] Cooney M.T., Selmer R., Lindman A., Tverdal A., Menotti A., Thomsen T., DeBacker G., Bacquer D.D., Tell G.S., Njolstad I. (2015). Cardiovascular risk estimation in older persons: SCORE O.P. Eur. J. Prev. Cardiol..

[25] May S., Hosmer D.W. (2004). A Cautionary Note on the Use of the Grønnesby and Borgan Goodness-of-Fit Test for the Cox Proportional Hazards Model. Lifetime Data Anal..

[26] Pennells L., Kaptoge S., White I.R., Thompson S.G., Wood A.M., Robert W., Tipping R.W., Folsom A.R., Couper D.J., Emerging Risk Factors Collaboration (2014). Assessing Risk Prediction Models Using Individual Participant Data From Multiple Studies. Am. J. Epidemiol..

[27] Hippisley-Cox J., Coupland C., Robson J., Sheikh A., Brindle P. (2009). Predicting risk of type 2 diabetes in England and Wales: Prospective derivation and validation of QDScore. BMJ.

[28] Seward J.P., Larsen R.C. (2007). Occupational Stress.

[29] Persechino B., Fontana L., Buresti G., Rondinone B.M., Laurano P., Fortuna G., Valenti A., Iavicoli S. (2017). Collaboration of occupational physicians with national health system and general practitioners in Italy. Ind. Health.

[30] Persechino B., Fontana L., Buresti G., Rondinone B.M., Laurano P., Imbriani M., Iavicoli S. (2016). Professional activity, information demands, training and updating needs of occupational medicine physicians in Italy: National survey. Int. J. Occup. Med. Environ. Health.

[31] Chiuve S.E., Cook N.R., Shay C.M., Rexrode K.M., Albert C.M., Manson J.A.E., Willett W.C., Rimm E.B. (2014). Lifestyle-based prediction model for the prevention of CVD: The healthy heart score. J. Am. Heart Assoc..

[32] Fernández-Labandera C., Calvo-Bonacho E., Valdivielso P., Quevedo-Aguado L., Martínez-Munoz P., Catalina-Romero C., Ruilope L.M., Sánchez-Chaparro M.A. (2019). Prediction of fatal and non-fatal cardiovascular events in young and middle-aged healthy workers: The IberScore model. Eur. J. Prev. Cardiol..

[33] Halaris A. (2013). Co-Morbidity between Cardiovascular Pathology and Depression: Role of Inflammation. Inflamm. Psychiatry.

[34] Cohen B.E., Edmondson D., Kronish I.M. (2015). State of the Art Review: Depression, Stress, Anxiety, and Cardiovascular Disease. Am. J. Hypertens..

[35] Hert M.D., Detraux J., Vancampfort D. (2018). The intriguing relationship between coronary heart disease and mental disorders. Dialogues Clin. Neurosci..

[36] Serra C., Rodriguez M.C., Delclos G.L., Plana M., López L.I.G., Benavides F.G. (2007). Criteria and methods used for the assessment of fitness for work: A systematic review. Occup. Environ. Med..

[37] Arpaia P., Cuocolo R., Donnarumma F., Esposito A., Moccaldi N., Natalizio A., Prevete R. (2021). Conceptual design of a machine learning-based wearable soft sensor for non-invasive cardiovascular risk assessment. Meas. J. Int. Meas. Confed..

[38] Persechino B., Fontana L., Buresti G., Fortuna G., Valenti A., Iavicoli S. (2019). Improving the job-retention strategies in multiple sclerosis workers: The role of occupational physicians. Ind. Health.

